# Molecular prevalence and phylogenetic characterization of *Plasmodium juxtanucleare* in Thai native chickens and fighting cocks across Kalasin Province, Northeast Thailand

**DOI:** 10.14202/vetworld.2026.1459-1469

**Published:** 2026-04-12

**Authors:** Jiranan Insee, Sirikanda Thanasuwan

**Affiliations:** 1 Department of Animal Science, Faculty of Agricultural Technology, Kalasin University, Kalasin, Thailand; 2 Department of Veterinary Technology, Faculty of Agricultural Technology, Kalasin University, Kalasin, Thailand

**Keywords:** avian malaria, *COXIII* gene, molecular prevalence, phylogenetic analysis, *Plasmodium juxtanucleare*, poultry parasite, Thailand, Thai native chicken

## Abstract

**Background and Aim:**

Avian malaria caused by *Plasmodium* spp. is an important vector-borne disease affecting poultry production in tropical and subtropical regions. Thai native chickens (*Gallus gallus domesticus*) and fighting cocks represent valuable genetic and economic resources in Thailand, yet information on the molecular epidemiology of avian malaria in northeastern Thailand remains limited. Kalasin Province contains diverse ecological environments with freshwater reservoirs, irrigated agricultural systems, and backyard poultry farming, which may facilitate transmission of haemosporidian parasites. Molecular tools targeting mitochondrial genes provide higher sensitivity than microscopic examination and allow accurate phylogenetic characterization. This study aimed to determine the molecular prevalence of *Plasmodium* spp., compare infection rates between free-range Thai native chickens and intensively managed fighting cocks, and analyze the phylogenetic relationships of circulating parasites using the mitochondrial cytochrome c oxidase subunit III (*COXIII*) gene.

**Materials and Methods:**

A cross-sectional survey was conducted from January to April 2025 using 181 blood samples collected from Thai native chickens (n = 112) and fighting cocks (n = 69) across 18 districts of Kalasin Province, Thailand. Genomic DNA was extracted using a commercial kit, and *Plasmodium* infection was detected by polymerase chain reaction targeting the mitochondrial *COXIII* gene. Positive amplicons were sequenced, and phylogenetic relationships were reconstructed using the Maximum Likelihood method. Differences in prevalence between host types and districts were evaluated using the Chi-square test, with p < 0.05 considered significant.

**Results:**

The overall molecular prevalence of *Plasmodium* spp. infection was 62.98% (114/181). Infection was higher in Thai native chickens (67.85%) than in fighting cocks (55.07%), but the difference was not statistically significant (p > 0.05). In contrast, prevalence varied significantly among districts (p < 0.05), ranging from 20% to 100%. Sequencing analysis revealed two haplotypes of *Plasmodium*. Phylogenetic analysis showed that all isolates clustered within Clade A and shared 99%–100% nucleotide identity with *Plasmodium juxtanucleare*. Haplotype I was dominant and detected in multiple chicken breeds, whereas Haplotype II formed a minor lineage closely related to *P. relictum*.

**Conclusion:**

This study confirms the hyper-endemic circulation of *P. juxtanucleare* in Kalasin Province and demonstrates genetic diversity within the local parasite population based on *COXIII* gene analysis. Similar infection rates in free-range and intensive systems indicate that environmental vector exposure plays a major role in transmission. These findings highlight the importance of molecular surveillance and vector control strategies and provide baseline data for future One Health studies on avian haemosporidian infections in Thailand.

## INTRODUCTION

Poultry farming plays a crucial role in global food security by providing an affordable and rapid source of high-quality animal protein compared to large livestock. In this sector, Thailand has established itself as a leading global hub, ranking fifth in the world for poultry meat production. Among the diverse poultry populations, Thai native chickens (*Gallus gallus domesticus*) hold unique socio-economic significance [1]. These indigenous birds are deeply integrated into Thai culture, serving purposes ranging from household food consumption and religious ceremonies to traditional sports, particularly cockfighting [2, 3]. They are highly valued by consumers for their superior meat quality, distinctive texture, and health-promoting characteristics [4–6]. However, despite their economic and cultural importance, these chickens can act as reservoir hosts for various infectious diseases when adequate preventive measures are not implemented on farms.

Avian malaria, caused by parasites of the genus *Plasmodium* (Haemosporida, Plasmodiidae) [7], is one of the most important blood protozoan infections affecting poultry production systems [8]. *Plasmodium gallinaceum* and *Plasmodium juxtanucleare* are the principal species infecting domestic chickens and wild fowl. Infection with these parasites is characterized by clinical signs such as anemia, lethargy, anorexia, ruffled feathers, and death [9]. The disease is highly prevalent in tropical regions, including Asia, East and South Africa, Central and South America, South Asia (*P. juxtanucleare*), and Southeast Asia (*P. gallinaceum*) [10]. These parasites are transmitted by hematophagous arthropod vectors, mainly mosquitoes (Family Culicidae) and biting midges (genus *Culicoides*, Family Ceratopogonidae). While *P. gallinaceum* is primarily transmitted by mosquitoes of the genera *Aedes*, *Culex*, and *Anopheles* [7], *P. juxtanucleare* is mainly transmitted by biting midges (*Culicoides* spp.). After an infected vector takes a blood meal, sporozoites invade reticuloendothelial tissues (hemopoietic system; exo-erythrocytic stage) and erythrocytes (erythrocytic stage), eventually developing into gametocytes circulating in the bloodstream [7, 11]. Some *Plasmodium* species cause more severe disease during the exo-erythrocytic stage in hepatic cells than in mammalian malaria [11]. Clinical manifestations in birds range from subclinical infection to severe anemia and death within one week, with reported mortality rates reaching 80%–90% [12].

In Thailand, molecular investigations of avian haemosporidians have received increasing attention in recent years. Several studies have reported the genetic diversity and prevalence of blood parasites in different avian hosts, including fighting cocks [13], backyard chickens (*G. g. domesticus*) [14], and wild birds in Chiang Mai Province [15]. Broader regional surveys have also described the distribution of these parasites throughout Southeast Asia [16]. Despite the growing body of evidence, information on the molecular epidemiology of *Plasmodium* spp. in northeastern Thailand remains limited. To date, no study has combined district-level spatial epidemiology with molecular phylogenetic characterization in this region. Unlike industrial poultry production areas, this province is characterized by widespread small-scale agriculture, where Thai native chickens are mainly raised under backyard conditions with minimal biosecurity, alongside intensively managed fighting cocks. In addition, the diverse topography of the province, including valleys, freshwater reservoirs, and areas near major dams, creates ecological conditions favorable for vector proliferation. Consequently, avian malaria represents a potential economic threat to local farmers due to unquantified productivity losses and chronic health problems in valuable birds.

The diagnosis of avian malaria has evolved from conventional microscopic examination to molecular techniques due to their greater sensitivity and specificity. Multi-locus analysis targeting the *cytochrome b*, cytochrome c oxidase subunit I (*COXI*), and *COXIII* genes provides high-resolution phylogenetic reconstruction [17]. However, using a single highly sensitive marker is often sufficient and more cost-effective for large-scale epidemiological surveys. The *COXIII* gene is a reliable mitochondrial marker because mitochondrial genomes occur in multiple copies within each parasite, resulting in improved detection sensitivity compared with single-copy nuclear genes [18]. Although *cyt b* remains the standard marker for lineage comparison in the MalAvi database, *COXIII* primers are effective for determining local prevalence and identifying dominant circulating species.

Despite the increasing number of molecular studies on avian haemosporidians in Thailand and neighboring countries, important gaps remain in the understanding of the epidemiology of *Plasmodium* infections in native poultry populations, particularly in northeastern Thailand. Previous investigations have mainly focused on limited geographic areas, specific bird species, or single farming systems, which restrict the evaluation of regional transmission dynamics. In addition, most available studies have relied on microscopic examination or a limited number of molecular targets, whereas information from the mitochondrial *COXIII* gene remains scarce in this region. Because mitochondrial genes are present in multiple copies within the parasite, the *COXIII* gene may provide greater sensitivity for detecting low-level infections and improve the identification of circulating parasite lineages.

Another important limitation of previous reports is the lack of studies integrating spatial epidemiology with molecular phylogenetic analysis across multiple districts within the same province. Kalasin Province represents a unique ecological setting characterized by freshwater reservoirs, irrigation systems, and mixed poultry production, which may favor the proliferation of mosquito and biting midge vectors. Furthermore, Thai native chickens are commonly raised under free-range conditions, whereas fighting cocks are generally kept under more controlled management, resulting in different levels of exposure to insect vectors. However, comparative molecular data between these two management systems are still lacking. This lack of evidence limits understanding of how environmental conditions, husbandry practices, and host type influence the transmission of *P. juxtanucleare* and other avian malaria parasites in endemic areas. Therefore, comprehensive molecular surveillance covering multiple districts and contrasting poultry management systems is needed to clarify the epidemiological status and genetic diversity of these parasites in northeastern Thailand.

Therefore, the present study was designed to investigate the molecular epidemiology of *Plasmodium* infection in Thai native chickens (*G. g. domesticus*) and fighting cocks raised in Kalasin Province, Thailand. The objectives of this study were to determine the molecular prevalence of *Plasmodium* spp. using polymerase chain reaction targeting the mitochondrial *COXIII* gene, to compare infection rates between free-range native chickens and intensively managed fighting cocks, and to evaluate the spatial distribution of infection across all districts of the province. In addition, nucleotide sequencing and phylogenetic analysis were performed to identify the circulating parasite species and to assess their genetic relationships with previously reported isolates.

By combining district-level sampling, comparison of host management systems, and molecular phylogenetic characterization based on the *COXIII* gene, this study provides baseline data on the distribution and genetic diversity of *P. juxtanucleare* in northeastern Thailand. The findings are expected to improve understanding of avian malaria transmission under tropical farming conditions and to support the development of more effective surveillance and control strategies for vector-borne infections in native poultry populations.

## MATERIALS AND METHODS

### Ethical approval

The study protocol was reviewed and approved by the Institutional Ethical Committee of Kalasin University, Thailand (approval no. KSU-AE-029; approved on 05 August 2024). All procedures involving animals were conducted in accordance with institutional guidelines for animal care and use and complied with the national regulations for animal welfare in Thailand.

Blood samples were collected from Thai native chickens and fighting cocks using standard veterinary procedures designed to minimize pain, stress, and discomfort. No experimental infection, surgical intervention, or harmful manipulation of animals was performed during the study. All sampling procedures were carried out by trained personnel under appropriate handling conditions to ensure animal safety.

Permission for sample collection was obtained from farm owners before sampling, and informed verbal consent was secured for the use of samples for research purposes. The study involved only routine diagnostic sampling, and therefore, no animals were sacrificed specifically for this research.

All laboratory procedures were performed in accordance with biosafety guidelines for handling biological samples, and appropriate measures were taken to prevent contamination and ensure safe disposal of biological waste.

The study was conducted in compliance with internationally accepted principles for ethical use of animals in research and followed the recommendations for humane treatment of animals in veterinary and biomedical investigations.

### Study period and location

**Figure 1:**
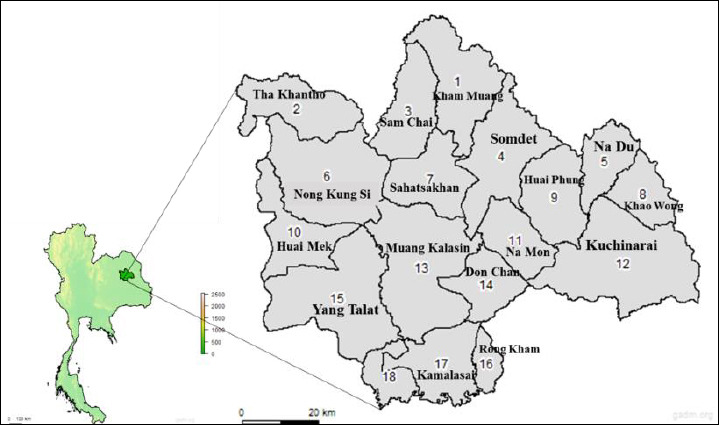
Map of Kalasin Province, Thailand (left), and the 18 districts of the sampling sites for chicken blood samples (right). The map was generated by modifying the Kalasin map from GADM version 2.8.

A cross-sectional study was conducted from January to April 2025, a period marking the transition from the cool dry season to the hot season, in Kalasin Province, Thailand (Figure 1). Kalasin occupies an area of 6,947 km² and is geographically located at 16.635540°N, 103.772418°E. The study area is situated at an altitude of approximately 147 m above mean sea level, with an average temperature of 26.8°C and annual rainfall of 1,407 mm. Blood samples were collected from all 18 districts of the province, namely Kham Muang, Tha Khantho, Sam Chai, Somdet, Na Khu, Nong Kung Si, Sahatsakhan, Khao Wong, Huai Phung, Huai Mek, Na Mon, Kuchinarai, Muang Kalasin, Don Chan, Yang Talat, Rong Kham, Kamalasai, and Kong Chai. The identification procedures were performed at the Laboratory of the Department of Veterinary Technology, Faculty of Agricultural Technology, Kalasin University.

### Sample size

Samples from 181 Thai native chickens and fighting cocks were collected from all 18 districts. The sample size was determined using the following equation:

Sample size = (1.96² pq) / L²

Here, n = sample size, p = expected prevalence, q = 1 − p, and L = limit of error on the prevalence (0.05). Since the actual prevalence in the Thai native chicken population was unknown but expected to be high based on regional data, the calculation was performed using an assumed prevalence of 90% (p = 0.9, q = 0.1). Based on this calculation (Z = 1.96 for 95% confidence interval), the minimum required sample size was 139 chickens [19, 20].

### Sampling

Farms were selected using convenience sampling based on the availability of animals and the willingness of owners to participate. A total of 181 blood samples were collected from Thai native chickens (n = 112) and fighting cocks (n = 69) within the study area described above. Blood samples (approximately 0.1–0.5 mL) were collected from the wing vein into sterile tubes containing ethylenediaminetetraacetic acid (EDTA) anticoagulant. Samples were placed in an ice box during transport to the Laboratory of the Department of Veterinary Technology, Faculty of Agricultural Technology, Kalasin University, and stored at −20°C before DNA extraction.

### DNA extraction and molecular examination of malaria infections

Genomic DNA was extracted from 200 µL of each blood sample using GF-1 Blood DNA Extraction Kit (Vivantis Technologies, Selangor, Malaysia) according to the manufacturer’s instructions. Genomic DNA was eluted in 50 µL of elution buffer. The concentration and purity of the extracted DNA were measured using a spectropho-tometer (NanoDrop, Thermo Scientific, Waltham, MA, USA). Only DNA samples with an A260/A280 ratio between 1.8 and 2.0 were used for further polymerase chain reaction analysis. Each extracted DNA sample was stored at −20°C until molecular identification of *Plasmodium* spp. was performed.

### Molecular detection by polymerase chain reaction (PCR)

A fragment of 377 bp of the mitochondrial *COXIII* gene of *Plasmodium* spp. was amplified using two primers, PMF (5′-CCTCACGAGTCGATCAGG-3′) and PMR (5′-GGAAACCGGCGCTAC-3′) [21]. Although this primer set has been reported to detect other haemosporidians, the present study specifically focused on *Plasmodium* spp.

**Figure 2:**
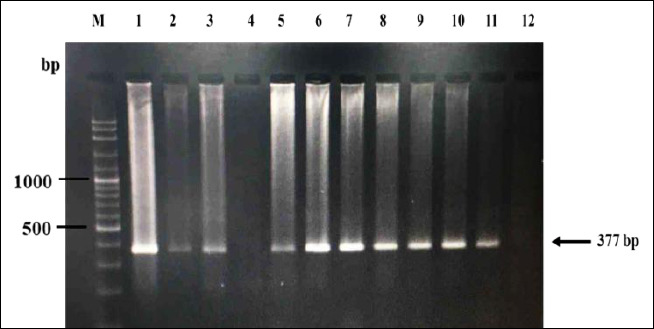
Agarose gel electrophoresis (1.5%) showing polymerase chain reaction amplification products of the mitochondrial cytochrome c oxidase subunit III (*COXIII*) gene (377 bp) of *Plasmodium* spp. Lane M: 100 bp DNA ladder; Lane 12: Negative control (nuclease-free water); Lane 1: Positive control (DNA from *Plasmodium juxtanucleare*-infected chicken); Lanes 2–11: Representative field blood samples. Lanes showing a band at 377 bp indicate positive infection.

The PCR was performed in a final volume of 25 µL containing 1.5 mM MgSO_4_, 0.2 mM dNTPs, 1× PCR buffer, 1 U of *Taq* polymerase (Vivantis Technologies), 0.2 µL of each primer (10 µM), and 2 µL of template DNA (10–50 ng). The amplification protocol consisted of an initial denaturation at 95°C for 5 min, followed by 35 cycles of denaturation at 95°C for 30 s, annealing at 59°C for 90 s, extension at 72°C for 30 s, and a final extension at 72°C for 10 min. All reactions included one negative control (ddH_2_O) for each batch of samples. Amplification products (5 µL) were separated on 2% agarose gel stained with ViSafe nucleic acid stain (Vivantis Technologies) and visualized using Gel Doc™ XR+ imaging system with Image Lab™ software (Bio-Rad, Hercules, CA, USA) under ultraviolet light to confirm positive amplification (Figure 2).

### Quality control and prevention of contamination

To ensure the reliability of the PCR assay, strict quality control measures were applied. Genomic DNA extracted from a chicken previously confirmed to be infected with *P. juxtanucleare* by sequencing was used as the positive control, whereas nuclease-free water was used as the negative control in every run. DNA extraction, master mix preparation, and gel electrophoresis were performed in separate areas to prevent cross-contamination. Filtered pipette tips were used throughout the procedure. Samples showing unclear bands were tested again in duplicate to confirm the results.

### Nucleotide sequencing and phylogenetic analysis

Thirty-two PCR amplicons showing clear bands of the target gene (377 bp) were selected for nucleotide sequencing (Macrogen, Seoul, South Korea). Chromatograms were checked visually, and sequences were edited and aligned using BioEdit 7.2 [22] and MEGA X software [23]. Sequence identity was verified using the BLAST tool of the National Center for Biotechnology Information. All sequences were deposited in the GenBank database under accession numbers PQ783679–PQ783710.

Before phylogenetic analysis, all sequences were examined in BioEdit software, and low-quality nucleotide regions at the 5′ and 3′ ends were removed. Alignment was performed using the ClustalW algorithm, and gaps or missing data were treated using the complete deletion option. The final dataset contained a uniform sequence length of 377 bp for all isolates. The best-fit nucleotide substitution model was selected based on the lowest Bayesian Information Criterion value using MEGA-XII. Phylogenetic trees were constructed using the Maximum Likelihood method, and branch support was evaluated with 1,000 bootstrap replications. Branch lengths were expressed as the number of substitutions per site.

### Statistical analysis

Statistical analysis was performed using SPSS version 25.0 (IBM Corp., NY, USA). The prevalence of *Plasmodium* spp. infection was expressed as a percentage. The Chi-square test (χ²) was used to compare infection rates between the two chicken management systems (Thai native chickens vs. fighting cocks) and among different districts. Differences were considered statistically significant when p < 0.05.

## RESULTS

### Prevalence of *Plasmodium* infection in native chickens

Molecular detection using PCR targeting the non-coding region of mtDNA revealed that 114 out of 181 samples were positive for *Plasmodium* spp., representing an overall prevalence of 62.98%. Regarding chicken management systems, the prevalence in free-range Thai native chickens was 67.85% (76/112), which was higher than in intensively managed fighting cocks (55.07%, 38/69). However, statistical analysis showed no significant difference between these two groups (χ² = 2.470, p = 0.116) (Table 1).

**Table 1:** Prevalence of *Plasmodium* spp. infection compared between chicken management systems.

Management System	Total Samples (n)	No. Positive	Prevalence (%)	95% CI
Thai native chicken (Free-range system)	112	76	67.86	58.75 – 75.83
Fighting cock (Intensive care system)	69	38	55.07	43.32 – 66.28
Total	181	114	62.98	55.77 – 69.65

χ² = 2.470, p-value = 0.116ns. ns = no statistical significance (p > 0.05), CI = Confidence interval.

In contrast, the spatial distribution of infection among the 18 districts in Kalasin Province showed statistically significant variation (χ² = 40.416, p = 0.001) (Table 2). The highest prevalence was observed in Kham Muang District (100%), followed by Huai Mek (90%) and Nong Kung Si (85.71%). Conversely, the lowest prevalence rates were found in Na Mon and Kamalasai (20%). Detailed prevalence data for all districts are presented in Table 2.

**Table 2:** Prevalence of *Plasmodium* spp. infection in Thai native chickens and fighting cocks across 18 districts in Kalasin Province.

District	Total Samples (n)	No. Positive	Prevalence (%)	95% CI
Kham Muang	10	10	100.00	72.25 – 100.00
Huai Mek	10	9	90.00	59.58 – 98.21
Nong Kung Si	7	6	85.71	48.69 – 97.43
Kong Chai	7	6	85.71	48.69 – 97.43
Khao Wong	6	5	83.33	43.65 – 96.99
Rong Kham	10	8	80.00	49.02 – 94.33
Tha Khantho	10	8	80.00	49.02 – 94.33
Na Du	10	8	80.00	49.02 – 94.33
Yang Talat	7	5	71.43	35.89 – 91.78
Muang Kalasin	20	14	70.00	48.10 – 85.45
Don Chan	10	6	60.00	31.27 – 83.18
Kuchinarai	10	5	50.00	23.66 – 76.34
Sahatsakhan	14	7	50.00	26.80 – 73.20
Somdet	10	5	50.00	23.66 – 76.34
Huai Phung	10	4	40.00	16.82 – 68.73
Sam Chai	10	4	40.00	16.82 – 68.73
Na Mon	10	2	20.00	5.67 – 50.98
Kamalasai	10	2	20.00	5.67 – 50.98
Overall	181	114	62.98	55.77 – 69.65

χ² = 40.416 p-value = 0.001*, CI = Confidence interval.

* indicates statistical significance (p < 0.05). Comparison among 18 districts.

### Molecular detection of malaria infections

**Figure 3:**
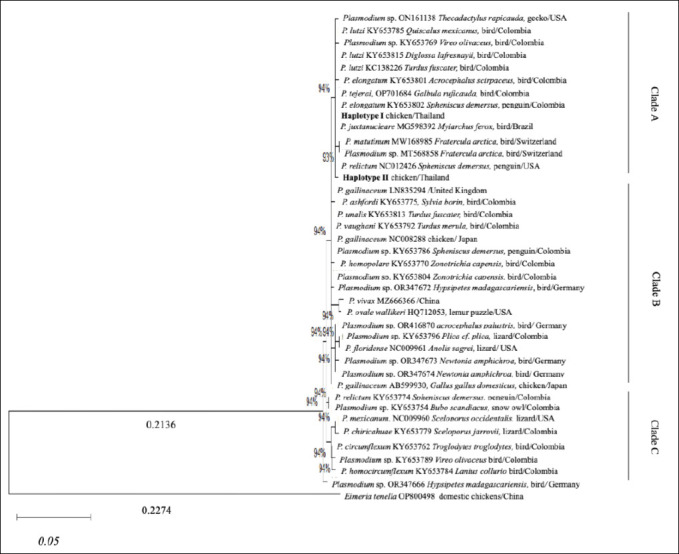
Phylogenetic tree constructed using the Maximum Likelihood method based on the mitochondrial *COXIII* gene sequences of *Plasmodium* spp. from Kalasin Province and reference sequences from GenBank. Bootstrap support values greater than 50% (based on 1,000 replicates) are shown at the branch nodes. The tree is drawn to scale, with branch lengths measured in the number of nucleotide substitutions per site (scale bar: 0.05). The Kalasin isolates (Haplotype I and II) clustered within Clade A, closely related to *Plasmodium juxtanucleare*.

Sequencing analysis of the 32 PCR products obtained from Kalasin Province revealed two distinct haplotypes of *Plasmodium* spp. circulating in the study area. BLASTn analysis indicated that the obtained sequences shared 99–100% nucleotide identity with reference *Plasmodium* sequences, predominantly *P. juxtanucleare*. The phylogenetic tree, constructed using the Maximum Likelihood method based on the mitochondrial *COXIII* gene, separated the analyzed sequences into three main clades (Clades A, B, and C). Notably, all isolates from Kalasin Province clustered within Clade A, grouping together with avian *Plasmodium* lineages, with *Eimeria tenella* (OP800498) used as the outgroup (Figure 3).

Haplotype I was the predominant lineage, identified in 29 samples. As detailed in Table 3, this haplotype exhibited a broad host range, being detected across all examined Thai native chicken breeds (Chee, Leung Hang Kaw, Dang, and local chickens) as well as in fighting cocks. Phylogenetically, Haplotype I formed a tight cluster with *P. juxtanucleare* (MG598392) from Brazil and *P. elongatum* (KY653802) from Colombia, supported by a strong bootstrap value of 94%. These results confirm that Haplotype I represents the pathogenic *P. juxtanucleare* lineage circulating in the study area.

**Table 3:** Distribution of *Plasmodium juxtanucleare* haplotypes among different Thai native chicken breeds and fighting cocks in Kalasin Province, with corresponding GenBank accession numbers.

Haplotype	Host/Breed	Sample ID	GenBank accession numbers	Closest NCBI Sequence (% similarity)
I	Thai Native Chicken - Chee	NM5, NM7, KR016	PQ783679–PQ783681	*P. juxtanucleare* MG598392 (99%–100%)
I	Thai Native Chicken - Leung Hang Kaw	MU21, U24	PQ783682–PQ783683	*P. juxtanucleare* MG598392 (99%–100%)
I	Thai Native Chicken - Dang	MU31, U32, MU37, K41	PQ783684–PQ783687	*P. juxtanucleare* MG598392 (99%–100%)
I	Thai Native Chicken - Local Chicken	RK43, K49, KW52, KW55, KW056, HM170, NS171, NS173, NS175, NC179, NC184, KC189	PQ783688–PQ783693, PQ783702–PQ783710	*P. juxtanucleare* MG598392 (99%–100%)
I	Fighting Cock	KS60, KS61, NK153, NK159, NK160, HM162, HM166, HM167	PQ783694–PQ783701	*P. juxtanucleare* MG598392 (99%–100%)
II	Thai Native Chicken - Local Chicken	NK152, NK157	PQ783696, PQ783698	*P. juxtanucleare* MG598392 (99%–100%)
II	Fighting Cock	KW002	PQ783690	*P. juxtanucleare* MG598392 (99%–100%)

In contrast, Haplotype II was detected in only three samples (local chickens and fighting cocks) (Table 3). Interestingly, this haplotype formed a distinct sub-branch within Clade A. Although still belonging to the broader avian *Plasmodium* group, it showed closer phylogenetic affinity to *P. relictum* (NC012426) and *P. matutinum* (MW168985) (bootstrap value = 93%) rather than clustering directly with the main *P. juxtanucleare* lineage. Clades B and C consisted of other reference *Plasmodium* species (e.g., *P. gallinaceum*, *P. vivax*) and did not contain any sequences from the present study.

## DISCUSSION

### Prevalence and epidemiological significance

In the present study, we examined the molecular prevalence and genetic diversity of *Plasmodium* spp. in Thai native chickens and fighting cocks in Kalasin Province. The overall prevalence detected by PCR was 62.98% (114/181), indicating a hyper-endemic status in this area. This finding is consistent with high infection rates reported across mainland Southeast Asia, including neighboring provinces in Thailand and Myanmar (86.8%–100%) [24, 25], backyard chickens (64.91%) [16], fighting cocks (88.76%) [13], chickens (85%) [26], and Burmese red junglefowls (70.59%) [9]. However, the prevalence observed in the present study is higher than that reported in insular regions such as Indonesia (30%) [27], wild raptors (9%) [28], and Thai native fowls (50%) [10].

Recently, *P. knowlesi* and *P. juxtanucleare* were detected in the salivary glands of Anopheles latens in southern Thailand [16]. In northern Thailand, a report described two cases in free-ranging wild birds and caged birds, showing that wild birds had a higher infection rate, possibly because of greater exposure to insect vectors [14]. In addition, *P. juxtanucleare* and *P. gallinaceum* were detected in biting midges (Diptera: Ceratopogonidae) in Thailand [3]. Variation in the prevalence of malaria infection among poultry species in Thailand may be due to differences in host susceptibility, presence of competent vectors, variation in vector species or strains, and differences in exposure to vectors [13]. Some studies have attempted to identify transmission-blocking activities against *P. gallinaceum*, showing that intramuscular injection of low doses of artesunate could block gametocyte production and transmission to the mosquito vector *Aedes aegypti* [29]. However, many farms in Thailand lack adequate vector control measures, do not prevent mosquito exposure, and may allow reservoir hosts to retain parasites in the blood for prolonged periods before clearance [13]. Rather than reflecting methodological differences alone, these regional variations likely indicate the influence of macro-ecological factors, such as the abundance of dipteran vectors in the Mekong region, on disease transmission intensity [30]. Consequently, because of their continuous exposure to insect vectors in open environments, Thai native chickens may serve as sentinel hosts for monitoring the circulation of haemosporidian parasites in tropical agroecosystems, reflecting real-time transmission risk at the poultry–vector–environment interface.

### Effect of management system on infection rate

A critical aspect of this investigation was the direct molecular comparison between poultry types reared under different management systems. Unlike studies focusing on a single host type, this approach allows evaluation of the influence of husbandry practices on disease exposure. Although the difference was not statistically significant (p > 0.05), a higher prevalence was observed in free-range Thai native chickens (67.85%) compared with intensively managed fighting cocks (55.07%).

This trend may be explained by differences in vector exposure related to management practices. Fighting cocks are high-value animals and are usually raised with strict protective measures. They are often kept in cages covered with mosquito nets or cloth during the night, which reduces exposure to *Culicoides* and *Anopheles* vectors during peak feeding periods. In contrast, free-range native chickens frequently roost outdoors or in open shelters, increasing the likelihood of repeated vector bites. These findings support the hypothesis that physical barriers and management practices strongly influence infection dynamics, potentially more than intrinsic host immunity [13, 31].

### Influence of environmental factors

Environmental heterogeneity also played a major role in disease distribution, as evidenced by statistically significant variation in prevalence across the 18 districts (p < 0.05), ranging from 20% to 100%. High prevalence areas such as Kham Muang (100%) are located near large freshwater reservoirs and irrigation systems associated with the Lam Pao Dam. This observation is consistent with previous studies showing that dam construction and irrigation systems can increase malaria risk by creating permanent breeding habitats for mosquito vectors such as *Anopheles* spp. [32].

These water resources, together with stagnant water, rice fields, and dense vegetation, provide suitable breeding conditions for *Culicoides* biting midges and *Anopheles* mosquitoes, which are the primary vectors of *P. juxtanucleare* and *P. gallinaceum* in Thailand [3, 33]. Although sampling in the present study was conducted during the dry season (January–April), the high prevalence observed in districts such as Kham Muang suggests that permanent water bodies associated with irrigation systems enable vector populations to persist year-round [29].

### Pathogenicity and host–parasite relationship

*P. juxtanucleare* is generally considered less pathogenic than *P. gallinaceum* and typically produces chronic infections rather than acute mortality [34]. Consistent with this, despite the high infection rate (62.98%), most PCR-positive chickens in the present study were asymptomatic or showed only mild clinical signs. This finding differs from previous reports describing morbidity in laying hens in southern Thailand [16] and clinical anemia in northern Thailand [14].

The absence of severe clinical signs may indicate endemic stability or long-term adaptation between native chicken breeds and circulating *P. juxtanucleare* strains. In such situations, native chickens may act as tolerant reservoir hosts, maintaining parasite transmission without developing severe disease, whereas naïve or exotic breeds may show higher mortality. These findings highlight the importance of PCR-based surveillance, because clinical observation alone cannot detect asymptomatic carriers and may underestimate the true prevalence of infection in endemic regions [7].

### Phylogenetic characterization

This study provides the first molecular characterization of *P. juxtanucleare* in Kalasin Province. Phylogenetic analysis based on the mitochondrial COXIII gene placed all 32 sequenced isolates within Clade A. Haplotype I (29 isolates) was the dominant lineage and clustered closely with *P. juxtanucleare* isolates from Brazil [35] and *P. elongatum* from Colombia [31], with strong bootstrap support (94%). The presence of only two haplotypes despite the high prevalence suggests stable endemic transmission rather than insufficient sampling.

The high nucleotide similarity between Thai isolates and South American strains also indicates strong evolutionary conservation of the mitochondrial *COXIII* gene. As shown in Table 3, Haplotype I was detected in all examined chicken breeds, suggesting that it represents a generalist lineage capable of infecting multiple hosts. Haplotype II (three isolates) formed a separate branch within Clade A and showed closer similarity to *P. relictum* (bootstrap support 93%). This limited distribution may indicate a local strain variant or an ancestral lineage, although further sampling is required. These findings highlight the complexity of parasite evolution and suggest possible lineage diversification within the *P. juxtanucleare* population in Thailand.

### Study limitations

Several limitations should be considered when interpreting the results. First, the cross-sectional design provides only a single time-point assessment and does not allow evaluation of seasonal variation or incidence. Second, sampling was limited to the dry season (January–April), and prevalence may be higher during the rainy season when vector populations increase. Third, the study relied on a single-marker PCR assay based on the mitochondrial *COXIII* gene. Although sensitive for detection, this approach provides lower phylogenetic resolution than multi-locus analysis (e.g., combined with *cyt b*). Fourth, microscopic examination of blood smears was not performed, preventing estimation of parasitemia levels. Finally, entomological surveys and clinical performance data were not included. Future studies integrating vector ecology, host health, and economic impact within a One Health framework are recommended.

## CONCLUSION

The present study confirmed the high molecular prevalence of *Plasmodium* infection in Thai native chickens and fighting cocks in Kalasin Province, Thailand, with an overall prevalence of 62.98%, indicating a hyper-endemic transmission status in this region. Molecular detection based on the mitochondrial *COXIII* gene demonstrated that all sequenced isolates belonged to the avian malaria parasite *P. juxtanucleare*, with two haplotypes identified, of which Haplotype I was the dominant lineage circulating across multiple districts and host types. The absence of a statistically significant difference in prevalence between free-range native chickens and intensively managed fighting cocks suggests that environmental exposure to insect vectors plays a major role in disease transmission, whereas variation among districts highlights the influence of ecological factors such as irrigation systems, reservoirs, and vector abundance.

From a practical perspective, the high prevalence observed in this study indicates that Thai native chickens may act as important reservoir hosts for avian malaria parasites under tropical farming conditions. Improved vector control, better housing management, and reduction of standing water around poultry farms may help reduce transmission risk, especially in areas located near irrigation networks or freshwater reservoirs. The findings also demonstrate the usefulness of PCR-based surveillance using the *COXIII* gene for detecting subclinical infections that cannot be identified by clinical observation alone, emphasizing the need for molecular monitoring in endemic regions.

A major strength of this study is the inclusion of samples from all districts of Kalasin Province combined with molecular detection and phylogenetic analysis, providing comprehensive baseline data on the distribution and genetic diversity of *P. juxtanucleare* in northeastern Thailand. However, the cross-sectional design, sampling during the dry season, and the use of a single mitochondrial marker limit the ability to evaluate seasonal variation, vector dynamics, and deeper phylogeographic relationships. Future studies should include longitudinal sampling, multi-gene analysis, vector surveillance, and clinical performance evaluation to better understand host–parasite–environment interactions.

In conclusion, this study provides the first district-level molecular evidence of hyper-endemic circulation of *P. juxtanucleare* in Kalasin Province and highlights the importance of integrating molecular surveillance, farm management, and vector control strategies to reduce the impact of avian malaria in native poultry production systems. These findings contribute essential baseline information for future epidemiological and One Health studies on haemosporidian infections in tropical regions.

## DATA AVAILABILITY

The data generated during the study are included in the manuscript.

## AUTHORS’ CONTRIBUTIONS

JS: Sample collection, DNA extraction, formal analysis, visualization, and manuscript review. ST: Conceptualization, methodology, data curation, project administration, supervision, writing, review, and editing. Both authors have read and approved the final manuscript.
